# Kinetics of Supercritical Drying of Gels

**DOI:** 10.3390/gels4010003

**Published:** 2017-12-29

**Authors:** İbrahim Şahin, Yaprak Özbakır, Zeynep İnönü, Zeynep Ulker, Can Erkey

**Affiliations:** 1 Department of Chemical and Biological Engineering, Koç University, Rumelifeneri Yolu, Sarıyer, Istanbul 34450, Turkey; isahin@ku.edu.tr (İ.Ş.); yozbakir@ku.edu.tr (Y.Ö.); zinonu15@ku.edu.tr (Z.İ.); 2 Department of Pharmacy, Altınbaş University, Mahmutbey Dilmenler Caddesi, No: 26, Bağcılar, Istanbul 34217, Turkey; zeynep.ulkerdemir@altinbas.edu.tr

**Keywords:** aerogel, supercritical drying, kinetics, mass transfer, diffusion, spillover, expanded solvents, modeling

## Abstract

Supercritical drying of gels is considered as the most important step of aerogel production since it enables preservation of the three-dimensional pore structure which lead to unique material properties such as high porosity, low density, and large surface area. An understanding of the kinetics of supercritical drying is necessary to provide insight into material development, scale-up, and optimization of the aerogel manufacturing process. Thus, investigation of supercritical drying is gaining increased attention in recent years. This review paper covers the experimental considerations and techniques to study the kinetics of supercritical drying, fundamental mass transfer mechanisms during the drying process and modeling efforts to predict the drying kinetics for varying operating conditions and gel properties. Transport phenomena involving diffusion, convection, spillage by volume expansion, and axial dispersion are discussed by providing the fundamental equations and empirical correlations to predict transfer coefficients. A detailed review of literature covering experimental and theoretical studies on kinetics of supercritical drying is presented.

## 1. Introduction

An aerogel is a lightweight solid derived from a gel in which the liquid component of the gel has been replaced with air. Aerogels have low densities, high porosities, large surface areas, and open pore structures [1]. Due to its transparency and superior thermal insulation properties, silica aerogel has been the most widely investigated type of aerogel with the objective of developing silica aerogel panels as an alternative to glass windows. The high cost of obtaining such panels and their poor mechanical properties have prevented their widespread use. In the beginning of the century, Aspen Aerogels developed a new type of composite blanket by infusing glass fibers with silica aerogel and started production of these blankets on an industrial scale. This development has spurred interest in other types of aerogels for applications other than thermal insulation such as drug delivery [2], sensing [1], catalysis [3], and adsorption [4].

### 1.1. Drying Techniques of Wet Gels

**Figure 1:**
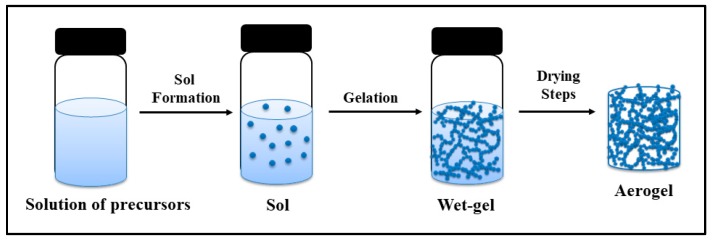
Schematic representation of sol-gel method to produce aerogels.

Aerogels are generally synthesized via sol-gel method involving sol formation, gelation, and drying steps, as illustrated in Figure 1. Precursors are allowed to react in an appropriate solvent, leading to formation of a sol which is a colloidal solution consisting of a suspension of very small solid particles. These particles crosslink or further polymerize and form a gel which has a highly porous structure in which the pores are filled with a liquid making more than 95% of the gel volume. Monolithic aerogels are obtained by gelation in appropriate molds [5], blankets are prepared by the addition of the sol solution to the fibrous matrix before gelation [6]. Gels in the form of particles can also be prepared by different techniques such as dripping method [7], emulsion-gelation method [8], and spraying [9]. Sometimes the gels are aged to strengthen their solid network. Subsequently, the liquid inside the gel is removed and replaced by air while preserving the original gel structure.

**Figure 2:**
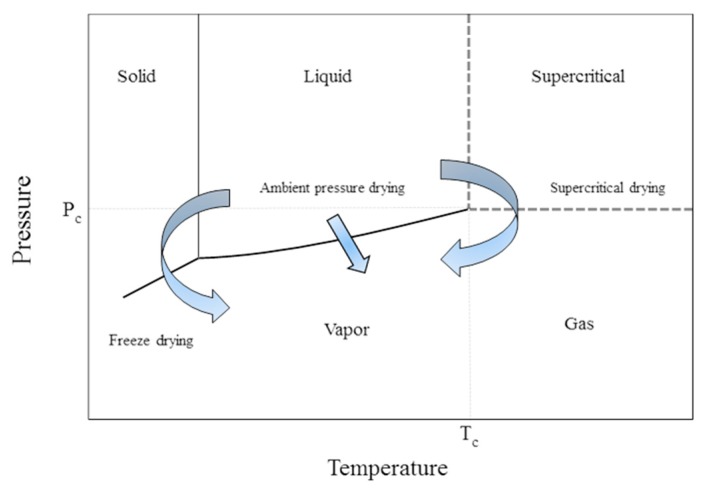
Phase diagram of a pure substance illustrating different drying routes. Adopted with permission from [10].

The removal of pore filling liquid should be performed while preserving the pore volume and matrix structure, hence, preserving the desired properties of the aerogels. Thus, this is the most critical step in aerogel production. There are three common methods for drying of gels, as shown in Figure 2:

- ambient pressure drying with matrix strengthening
- freeze drying
- supercritical drying

### 1.2. Ambient Pressure Drying with Matrix Strengthening

**Figure 3:**
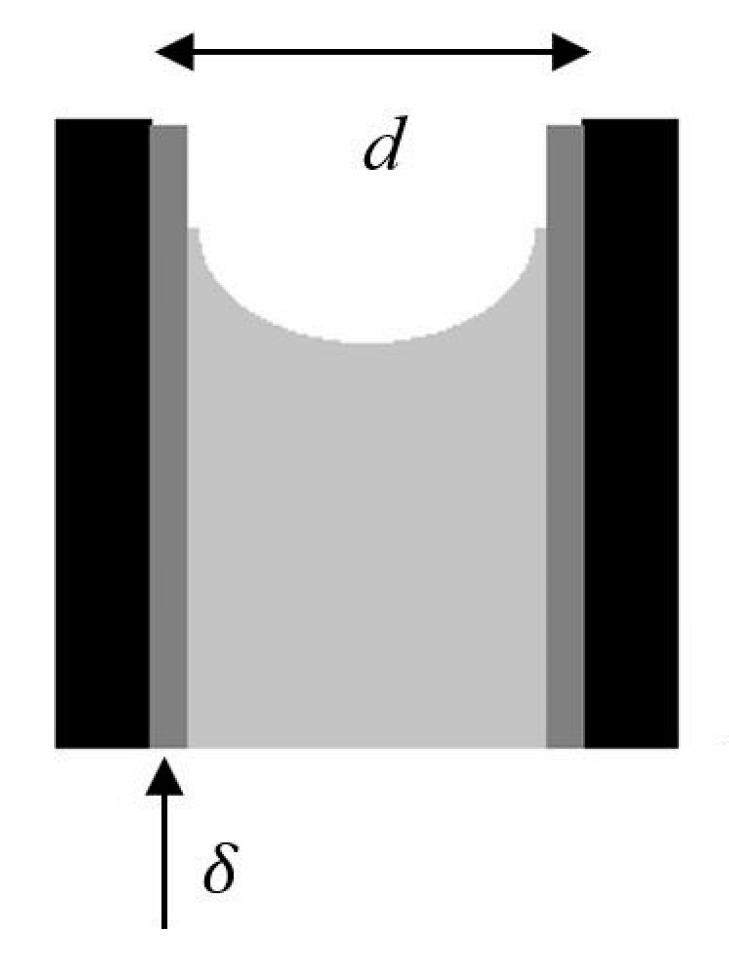
Illustration of a meniscus in a pore of wet gel. Adopted with permission from [10].

Ambient pressure drying is the process of drying the wet gels by evaporating the pore liquid at ambient pressures and at temperatures ranging from room temperature to 200 °C [11]. If the wet gel is dried under ambient conditions without matrix strengthening, it can shrink significantly, losing its porous structure. The most important feature of aerogels, their nano-sized pores is also the main reason for this shrinkage. As the solvent evaporates from the pores of the gel network, the radius of curvature of vapor-liquid interface decreases, exerting a pressure on the gel surface. The maximum pressure caused by the resulting meniscus in a pore of diameter *d* can be calculated as follows for a completely wetting solvent [10]

(1) $$P_{cap}=-\frac{4\gamma_{LV}}{d-2\delta }$$

where $\gamma_{LV}$ is the surface tension of the vapor-liquid interface, *d* is the diameter of the pore, and δ is the thickness of the liquid layer adsorbed on the solid surface, as represented in Figure 3.

The fragile porous structure of the wet gels cannot withstand the capillary pressures induced upon solvent evaporation which can be as high as nearly a thousand bar for a pore with 5 nm diameter filled with water as seen in Table 1. The thickness of the adsorbed layer is around 1 nm and depends on temperature and vapor pressure of the solvent, as well as the strength of hydrogen bonding between the liquid and the solid [12,13].

Consequently, the gels crack or shrink. Moreover, pore size distribution within the porous network leads to an inhomogeneous distribution of the forces acting on the fragile porous gel structure, which also may cause the destruction of the network. One of the ways to reduce this high capillary pressure is to use a solvent which has a low interfacial surface tension [14]. However, since the pores are nano-sized, the pressure will still be large enough to damage the structure of the gel as shown in Table 1.

In order to use ambient pressure drying method and preserve the high pore volume of the matrix at the same time, synthesis steps are combined with either surface treatment [15] or network strengthening [16] or combining both for the “springback effect” which is the re-expansion of the wet gel [17].

### 1.3. Freeze Drying

Freeze drying is another technique which can be used to dry the wet gels [17]. In this process, the solvent inside the pores is frozen by lowering the temperature below the freezing point of the solvent. Subsequently, the pressure is reduced below the sublimation pressure at this temperature generally by pulling vacuum on the system. The solvent sublimes and is removed from the pores without the formation of a liquid–vapor interface. Once the solvent is removed, the system is pressurized, and the sample is brought back to room temperature. Aerogels that are obtained this way are sometimes called cryogels.

Freezing might damage the nanostructured gels as freezing could lead to the growth of crystals and development of stress inside the pores possibly leading to fracture of the matrix. If the solvent is water, the situation is more severe since water expands when it freezes which may destroy the pore structure. Moreover, if the solvent is an alcohol, another difficulty arises such as achieving the freezing temperature (*T_f_* of ethanol is 160 K as an example) [10]. There are also other disadvantages such as long aging periods to obtain a strong enough solid network or time consuming sublimation steps [10,17].

### 1.4. Supercritical Drying

**Figure 4:**
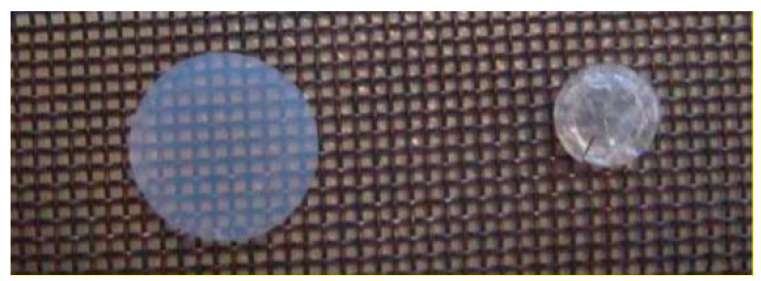
Silica gel images obtained by supercritical drying (**left**) and ambient drying (**right**). Reprinted with the permission from [21].

Supercritical drying which can be described as the extraction of the solvent from the pores of the gel with the use of supercritical fluids is another method which can be used for drying the wet gels. As shown in Figure 2, a fluid reaches its supercritical conditions when it is compressed and heated above its critical temperature and pressure. Supercritical fluids (SCFs) have liquid-like densities which enable them to function as solvents. Moreover, higher diffusion coefficients in supercritical fluids than in liquids combined with their gas like viscosities result in enhanced mass transfer characteristics. Supercritical drying generally yields aerogels which have higher pore volumes and porosities, higher surface areas when compared to freeze drying and ambient pressure drying [18,19,20]. A comparison of the two gels dried using supercritical CO_2_ and ambient drying is shown in Figure 4 which shows that shrinkage is significantly less (high porosity) with supercritical drying [21].

## 2. Supercritical Drying

**Figure 5:**
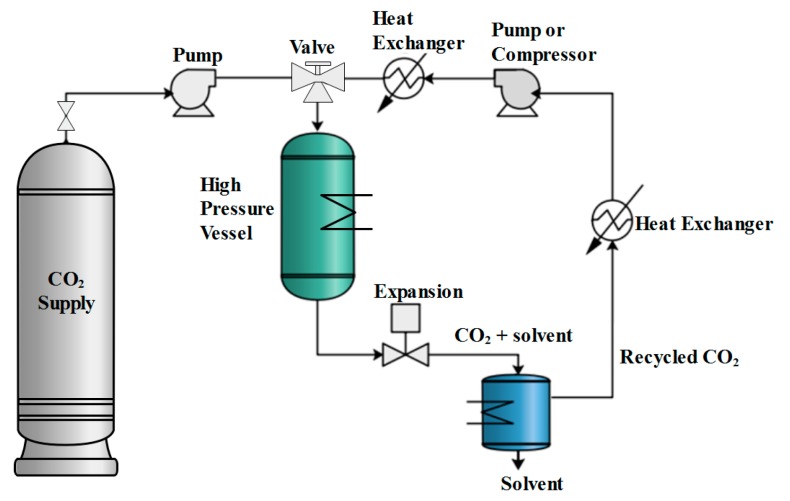
Flowsheet of a supercritical CO_2_ drying cycle.

A typical flow diagram of a continuous supercritical drying process is shown in Figure 5.

The gel is placed inside a high pressure vessel with an excess amount of solvent that is generally the pore liquid in the gel and the vessel is sealed. The vessel is then heated to the extraction temperature which is generally slightly above the critical temperature of CO_2_. Alternatively, the vessel is sometimes preheated before placing the gel in order to shorten the time to reach thermal equilibrium and prevent excessive evaporation. The vessel is then pressurized to the extraction pressure, which is higher than the critical pressure of the solvent-CO_2_ mixture. Subsequently, supercritical carbon dioxide (scCO_2_) is passed continuously over the aerogel, extracting the pore liquid. At the exit of the vessel, CO_2_-solvent mixture is expanded to a lower pressure leading to two phases: a vapor phase rich in CO_2_ and a liquid phase that is rich in solvent. The solvent rich phase is collected, and the CO_2_ rich stream is recycled back with a pump or compressor. The extraction is continued until solvent in the pores of the gel is removed completely. Then the system is depressurized slowly to prevent the formation of a vapor-liquid interface. Finally, the aerogel is taken out of the vessel after the pressure is reduced to one atmosphere.

The composition of the effluent stream from the extraction vessel changes as a function of time. Generally, a high drying rate is observed initially and the rate decreases as time goes on. The drying time can be regarded as the time where more than a certain percentage of the pore liquid is removed from the gel. This amount is governed by the application of the aerogel. In some cases such as production of silica aerogel based blankets, the residual solvent is removed by heating to elevated temperatures. The kinetics of supercritical drying of gels are important for:

1. Scaling up of laboratory scale drying units to pilot and industrial scale.
2. Assessing the economics of production of aerogels on an industrial scale.
3. Speeding up aerogel research in the laboratory by shortening drying times.
4. Providing information on the pore properties of aerogels such as porosity, pore volume, and tortuosity.
5. Improving mass transfer models for aerogels which can also be useful for impregnation of aerogels with a variety of chemicals and also for solvent exchange.
6. Providing information on the nature of capillary forces in aerogels.
7. Improving our understanding of convective mass transfer in SCFs and enable the development of correlations for convective mass transfer coefficients for SCFs.
8. Improving our understanding of axial dispersion in packed beds in SCFs.
9. Providing information on the effective diffusivities in aerogels.

At the end of the gelation process, the gel consists of a solid network in a solution. The solution can be aqueous or an organic solvent or a mixture of an organic solvent and water. Since water has limited solubility in scCO_2_, it cannot be extracted efficiently. Therefore, water is replaced with a solvent that has high solubility in scCO_2_. This solvent exchange sometimes leads to shrinkage of the gels due to solvent-gel interactions. Therefore, the type of solvent also is important to minimize this shrinkage [22].

The solvent replacing water in pores of the gel is selected considering its availability, toxicity, vapor pressure, and flammability which is very important for handling. It should be noted that even though the solvent has good interaction with the gel during solvent exchange which results in low shrinkage, it may not be very suitable for supercritical drying [22].

Solvents which are commonly used for this purpose are ethanol, acetone, and methanol due to their mild binary mixture critical temperatures with CO_2_. At 313.15 K, critical pressures of binary mixtures of CO_2_-ethanol, CO_2_-methanol, and CO_2_-acetone are around 8.0, 8.2, and 7.5 MPa, respectively [23,24]. Among these solvents, acetone is extremely flammable. Due to its low flash point of −20 °C, even at low fractions in air it is susceptible to explosions or flash fires. Such concentrations can occur if there is a large leak from the high pressure extraction vessel during drying. Although ethanol and methanol are also considered flammable with flash points of 16.6 and 12 °C, respectively, they can be used with caution. Vapor pressures of such solvents should also be considered since, as the solvent evaporates, its vapor is emitted into the environment, posing risks both for employees and for fire hazards. Ethanol is usually preferred due to its lower vapor pressure and methanol is considered toxic which is especially important for food and pharmaceutical applications. Moreover, the obstacle of shrinkage of gels with ethanol during the solvent exchange can be overcome by stepwise solvent exchange instead of immersion of the gel directly to pure ethanol [22]. Therefore, selection of ethanol is the best choice of solvents, especially when working with organic gels for health-related applications.

## 3. Kinetics of Supercritical Drying

### 3.1. Experimental Techniques to Investigate Drying Kinetics

The experimental protocols used for laboratory scale supercritical drying of gels vary widely among laboratories. The type of equipment varies from commercial supercritical extraction units to homemade units. It is not uncommon to charge extraction vessels with CO_2_ and leave the system for an extended period of time before starting the flow of CO_2_. In some systems, there are no pumps and high pressure CO_2_ in cylinders is just transferred into vessels and a certain amount of CO_2_ is discharged after a certain period of time. The vessel is then filled with fresh CO_2_ from the cylinder. The cycling goes on until the gel is dried. There are some reported studies in the literature where the drying of a single sample is continued for almost one week [11,25,26]. The experimental variables such as temperature, pressure, and flowrate are often set quite arbitrarily. The depressurization rate which may cause shrinkage and cracking if it is too high is also set quite arbitrarily and is unknown in many cases. In some vessels, CO_2_ bypasses the gels leading to incomplete drying and thus cracked gels. These factors significantly affect the speed and effectiveness of aerogel research projects as well as reproducibility of the produced aerogels both in the same laboratory and also among different laboratories.

The following items are present in typical laboratory equipment to investigate kinetics of continuous supercritical drying of gels.

- A CO_2_ feeding system consisting of a CO_2_ tank and a high pressure-pump to feed CO_2_.
- A temperature and pressure controlled high pressure vessel in which gel to be dried is placed, valves to start/stop the CO_2_ flow into the vessel and to adjust the flow rate, and pressure transducers and thermocouples to monitor operating conditions.
- A back pressure regulator or a needle valve to control the pressure in the vessel.
- Flowmeters to measure the flow rate of the inlet and/or exit streams.
- A system to measure the composition of the exit stream from the vessel as a function of time.

Researchers have used different types of pumps such as HPLC pump [27], membrane pump [28,29], recirculation gear pump [30], nitrogen-driven piston pump [31], air driven pneumatic pump [5,32,33], and syringe pump [34]. Among these, air driven pneumatic pumps are robust and trouble free. The pressure is set by using back pressure regulators or micro metering needle valves.

The high pressure vessel is the core component of the equipment in which extraction of pore filling solvent is carried out. Size and geometry of the vessel is determined based on the dimensions of the wet gel to be dried. Vessel geometry can be rectangular or cylindrical with volumes ranging from several milliliters to several liters depending on the amount of gel. Researchers should be careful about some important aspects to ensure proper and effective drying of the gel. First, there should not be any CO_2_ leakage during operation and a good contact between the scCO_2_ and the wet gel should be sustained. Channeling of scCO_2_ should be avoided. In the case of monolithic gel drying, the vessel geometry should allow a uniform velocity distribution over the entire body of the gel, eliminating spots with low CO_2_ mass flow. Therefore, flow distributors can be connected to the vessel entrance if necessary. These can be trays or packing material used in conventional applications involving packed beds. Besides, necessary measures should be taken to prevent any blockage in the exit lines connecting the high pressure vessel to the detecting system. We recommend the use of micron size filters, frits, or glass wool at the exit end of the vessel.

The next step is to fill the vessel with the gel. In most of the studies, excess solvent is added to the vessel along with the wet gel. This is necessary to prevent contact of the wet gel sample with air and evaporation of the solvent from the wet gel surface which might cause the shrinkage of the gel and lead to the formation of cracks. We observed such behavior when excess ethanol sufficient to cover the wet gels was not added. For example, for drying of alginate gels, a considerable portion of centimeter sized beads shrunk during drying when the gels were not placed in ethanol. In order to prevent the evaporation of the solvent from the gel, an amount necessary to saturate the gas in the free volume of the vessel can be added. However, the exact amount of excess solvent which would prevent the shrinkage is not very clear. A commonly used method is to cover the whole bed of gel particles or the whole body of a monolithic gel with solvent. After the wet gel samples are properly placed in the vessel and covered with excess solvent, the exit valve is opened, so that scCO_2_ can push the excess solvent out.

**Figure 6:**
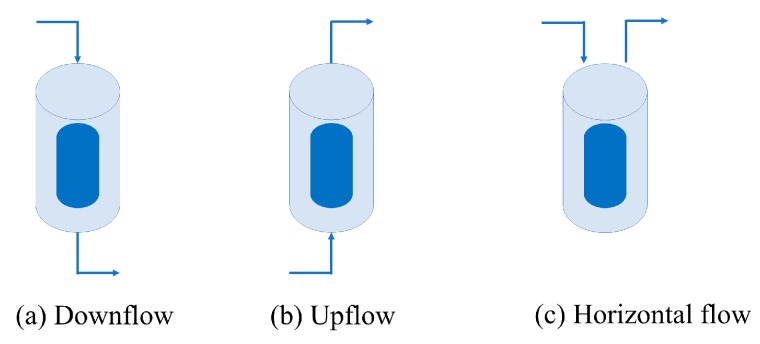
Different flow systems used in supercritical drying experiments. (**a**) Downflow; (**b**) upflow; (**c**) horizontal. Arrow directions show entrance of pure CO_2_ and exit of the effluent stream consisting of CO_2_ and the solvent whereas the dark blue cylinder represents the gel placed inside the vessel.

Another important consideration is the flow direction of scCO_2_ in the vessel. Figure 6 shows three different flow configurations such as downflow, upflow, and horizontal flow for a cylindrical vessel and gel material.

There are examples of downflow [5,29,30,31,35], upflow [28,32,33,36,37], and horizontal flow [27,34] systems in the literature used for laboratory scale supercritical drying. According to our own experience on drying experiments, we recommend the use of the downflow system. The main reason is the fact that density of the solvent (ethanol in most of the cases) is higher than the density of CO_2_ at typical temperatures and pressures employed in supercritical drying. Thus, difficulty of pushing the high density solvent by low density CO_2_ in the upflow direction elongates the drying time due to probable occurrence of back mixing. This would also create problems in evaluation and understanding of the concentration profiles measured during drying because of the probable back mixing of the diffused solvent from the pores of wet gel. The same problems are also present in the case of horizontal systems. However, in the case of downflow systems, solvent can be be pushed out of the vessel almost immediately with the charging of the vessel with high pressure CO_2_ without causing any back mixing of the solvent.

The last component of the drying equipment is the analytical device to measure the composition of the effluent stream. The selection of the analytical technique depends on many factors. For instance, drying of large monolithic gels requires several hours. However, drying of micro particles or beads can usually be carried out in less than an hour and demands a measuring system that is either continuous or uses very short time interval measurements. In either case, measurements should also be repeatable and the mass balance for the extracted pore liquid should be closed with an acceptable accuracy.

#### 3.1.1. Techniques Used to Measure Concentration of the Solvent in CO_2_

Techniques which are used to determine the concentration of the solvent in scCO_2_ can be divided into three categories:

- chromatography
- gravimetric determination
- in situ spectroscopy

Since most of the kinetic investigations were carried out on silica gel/ethanol as gel/solvent pair, the term “solvent” is used interchangeably with ethanol for the following paragraphs, unless otherwise stated.

Gas chromatography (GC) was used in some studies on kinetics of supercritical drying. Wawrzyniak et al. used a back pressure regulator after the extraction vessel to expand the effluent stream to atmospheric pressure [27,38]. This expanded stream was then passed through the sampling valve of a GC and analyzed every three minutes. Masmoudi et al. used a similar system for measurement of concentration of isopropyl alcohol as the solvent [39]. This system included a special capillary through which fluid samples near the gel were withdrawn every 1.5 min and injected into a micro gas chromatograph. The drawback of using gas chromatography is longer measurement intervals. The initial phase of the drying, in which a rapid decrease in the solvent amount inside the gel pores is generally observed, cannot be monitored well since sampling and analysis by a GC usually takes several minutes. However, it may be possible to use multiport sampling valves to take samples and analyze them later.

**Figure 7:**
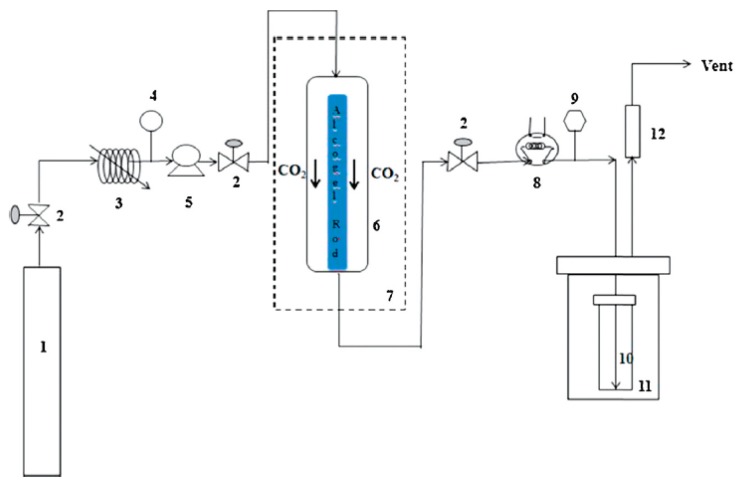
Schematic representation of process flow diagram of drying unit used in our studies (1. CO_2_ tank, 2. Valve, 3. Cooler, 4. Pressure transducer, 5. Pump, 6. Tubular extraction vessel, 7. Oven, 8. Micro-metering Valve, 9. Thermocouple, 10. Sample collection vial, 11. Dry ice cooling bath, 12. Rotameter). Adopted with permission from [5].

Griffin et al. measured the effluent flow rate using a Coriolis flow meter and connected a series of heated decompression valves, decreasing the pressure to near ambient pressures [31]. Then, the effluent was directed toward a tee-junction where liquid ethanol was drained and collected in a beaker which was placed on a continuously recording digital scale. The gaseous phase exiting the junction was fed to an infrared absorption-based hydrocarbon detector by which the concentration of the ethanol was measured. Since five decompression valves were used, control of the flow rate became difficult, especially at the initial period of drying. This led to noise like spikes in the measured concentration profiles and difficulty in closing mass balance with a low percent deviation. More commonly, a large number of researchers utilized a cold trap to capture the solvent with almost 100 percent efficiency [5,28,29,32,33,37]. Dry ice bath was used in several studies as the cooling medium decreasing the temperature of the collector vial down to −70 °C [5,33,37]. A needle valve was generally placed before the cold trap, enabling both adjustment of the gaseous phase flow rate and the decompression the effluent stream. Flow rate of CO_2_ rich gaseous phase was monitored by a rotameter and concentration of the solvent in this stream was measured by a using an alcohol meter in several studies [32,33]. Moreover, ethanol rich liquid phase was collected in glass vials within certain time intervals and weighed. Vials were changed in specific time intervals depending on the total time of the drying experiment. For instance, a period of one minute may be sufficient if the drying takes less than an hour. However, longer durations might be preferred for the case of longer drying times. Besides, time intervals in changing the vials may be short for the initial fast drying period, then, relatively longer intervals may be used for the later stages of drying. In doing so, all phases of drying can be monitored accurately. Calculations showed that use of dry ice bath captures almost all of the ethanol in the effluent, leading to mass balance closures with a small percent deviation [5,33,37]. Figure 7 shows the schematic of experimental set-up used in our laboratory consisting of a vial placed in a cooling bath as the separator unit.

Lastly, high pressure spectroscopy is a powerful tool which has been used to measure compositions of high pressure mixtures in a wide variety of studies by means of IR, UV, and NMR measurements [40]. In situ analysis of supercritical drying was studied by Quiño et al. utilizing a one dimensional Raman spectroscopy to reveal mass transport mechanisms taking place inside the porous network of the gel [34]. As a result, spatially resolved composition and concentration fields developing inside silica gels during drying were obtained. Although this technique was powerful, it required the installation of an optical set-up, use of a custom high pressure chamber, and data analysis tools. Thus, we strongly recommend this technique if the purpose of the experiment is to study fundamentals of transport mechanism and measure composition dependent diffusion coefficient. Recently, Baloch et al. developed an experimental technique to measure the composition of ethanol-CO_2_ mixtures at high pressures by frequency response of microcantilevers [41,42].

#### 3.1.2. Further Considerations

Last step of the experimental procedure is the depressurization of the extraction vessel. In this step, pores of the gel are initially filled with supercritical CO_2_. As the pressure is reduced, CO_2_ flows out of the pores slowly and supercritical CO_2_ becomes gaseous CO_2_ when the pressure is reduced below the critical pressure. Finally, gaseous CO_2_ is left as the pore filling gas at the end of the depressurization. View cell experiments showed that considerable shrinkage of the gel can happen during depressurization of the vessel [30]. This phenomenon can be attributed to mechanical stresses due to the expansion of CO_2_ inside pores. The origin of mechanical stresses was the rate at which scCO_2_ was depressurized. If the depressurization rate is too high, pressure outside of the gel will drop faster than inside since the pore filling CO_2_ will not be able to flow out of the complex pore network with the same rate [43]. A critical depressurization rate was observed in studies for silica gels such that, above this value, cracks appeared in the aerogel [30,44]. Besides, both pore size and porosity was found to change with the depressurization rate at high rates [45]. Thus, the depressurization rate should be selected carefully and reported along with the other experimental conditions. A slow rate of depressurization is recommended to have crack-free and less shrunk aerogels. However, slow depressurization would also lead to increased operating times. Therefore, a rate just below the critical value might be preferred. During the depressurization, the gel also cools due to the Joule-Thompson effect which may lead to the formation of a vapor-liquid interface. Therefore, slow depressurization rate under heating is also preferable in this regard.

Another important experimental consideration is the amount of solvent left inside the pores after supercritical drying. According to our observations, there is always a small portion of solvent left in the pores corresponding to 0.5–2% of total amount of the solvent extracted. This is due to the difficulty of removing the strongly adsorbed layer of solvent on the interior surface of the gel. This amount is determined gravimetrically by the weight difference of the aerogel just after supercritical drying and after further drying in oven. Generally, no shrinkage occurs during oven drying. However, solvent left can enter the two-phase region during depressurization for the case of insufficient drying leading to occurrence of cracks and shrinkage [37]. This solvent can be removed by subjecting the aerogel to high temperatures.

### 3.2. Mass Transfer Mechanisms in Supercritical Drying of Gels

When a wet gel is subjected to scCO_2_ stream, convective mass transfer of CO_2_ from the flowing scCO_2_ stream to the surface of the wet gel takes place [31,46,47]. The concentration gradient of pore liquid between the gel surface and the external scCO_2_ stream is high so that that a very high convective mass flux of the pore liquid from the gel surface to the flowing stream occurs. CO_2_ diffuses inside the pores towards the center of the gel and meanwhile, ethanol diffuses inside the pores towards surface of the gel. The volume of pore liquid can expand inside the pores due to dissolution of CO_2_ in the pore liquid leading to spillage of the pore liquid from the gel to scCO_2_ stream. The pore liquid is transferred by convective mass transfer with the flowing scCO_2_ stream. As drying progresses, the concentration gradient of the pore liquid between the surface and the flowing scCO_2_ stream decreases with time, which results in a decrease in convective mass flux of the pore liquid from the surface and thus a decrease in its extraction rate from the pores. As the process continues, the concentration of pore liquid decreases over time, whereas concentration of CO_2_ in the pores increases and eventually, almost all the liquid in the pores is replaced by scCO_2_.

#### 3.2.1. Diffusion

Diffusion in aerogels takes place through a cramped and tortuous interconnected open pore network of the aerogels [11,38,48]. The mass transfer flux of pore liquid, *N_a_*, by diffusion throughout the fluid-filled pores in aerogels is typically described by modified Fick’s Law [49]

(2) $$N_{a}=-D_{e}\frac{\partial C_{a}}{\partial x}+x_{a}\left(N_{a}+N_{b}\right)$$

where *D_e_* is the effective diffusion coefficient, *x_a_* is the mole fraction of the solvent species, $\partial C_{a}$/∂x is the concentration gradient. *D_e_* is less than the binary diffusion coefficient of pore liquid and CO_2_ (*D*_12_) due to the effects of aerogel porosity (*ε*) and tortuosity (*τ*) and it is usually given by [39,49]

(3) $$D_{e}=\frac{\left(D_{12}\varepsilon \right)}{\tau }$$

Diffusion coefficient strongly depends on the structural properties of the aerogels. Sometimes this can be corrected further by a term containing ($\lambda /d_{p}$) where λ is ratio of the size of the molecules and $d_{p}$ is the pore diameter [48,49]. Knudsen gas diffusion and molecular or bulk diffusion are significant diffusion mechanisms that occur in the aerogel pores. Aerogels have a pore size distribution, generally in the mesopore range and hardly in the micropore range, with pores typically ranging from 5 to 100 nm and an average pore diameter between 20 and 40 nm [11,48,50]. Diffusion in mesopores is mostly governed by molecular diffusion since the pore size is at least one order of magnitude larger than the mean free path of gas molecules. Knudsen diffusion takes place when the mean free path of the molecules is larger than the pore size so that the diffusing molecules collide more frequently with the pore walls than with the other diffusing species, which can be significant within the pores in the micropore range [48,49]. The overall diffusion coefficient (*D_t_*) in a binary mixture in aerogel considering *D*_12_ and Knudsen diffusivity (*D_k_*) is therefore calculated by [11,46]

(4) $$\frac{1}{D_{t}}≅\frac{1}{D_{12}}+\frac{1}{D_{k}}$$

(5) $$\frac{1}{D_{k}}=\frac{2}{3}r_{p}\sqrt{\frac{8RT}{\pi M}}$$

where $r_{p}$ is pore radius; *M* is molecular weight of CO_2_, kg/kmol; *R* is ideal gas constant, kJ/kmol; and *T* is temperature, K.

Aerogel porosity can be determined by several methods such as gas adsorption, mercury porosimetry, and light scattering [11,17,33,51]. Gas adsorption is the most widely utilized method to determine aerogel porosity using an inert gas, usually nitrogen, at its boiling point. The adsorbed nitrogen amount at saturation pressure is converted to liquid volume which is the pore volume. The Brunauer-Emmett-Teller (BET) equation is used to calculate specific surface area of aerogels from volume of the gas adsorbed at pressures up to around 0.2 atm [11,51]. At high partial pressure in the adsorption/desorption isotherms, the pore size distribution of the sample which is the distribution of pore volume with respect to pore size is determined according to Barrett-Joyner-Halenda (BJH) method through Kelvin equation. The gas adsorption methods are usually applicable for characterization of pores in the mesopore range. However, micropore volume of aerogels can be determined such as *t*-plot or Dubinin-Radushevich method. In mercury porosimetry technique which can be used to explore the pores between 3.5 nm and about 500 µm, mercury is forced into the aerogel porous structure to determine size of pores; however, this method may collapse the aerogel solid network due to the high compressive force [52,53]. The scattering is based on angle-dependent deflection of radiation within the aerogel due to the features such as solid network or pores. The small-angle radiation scattering, particularly X-ray and neutron, are well-suited to determine the fractal geometry of the aerogel pore network [11,17].

Aerogel tortuosity is typically used to characterize connecting pore channels that are not straight. It is therefore a simple number showing how much diffusion will be retarded in the porous network due to longer connecting pore channels [50,54,55]. Tortuosity of aerogels is typically taken as the reciprocal of porosity, but good methods are needed to determine the tortuosity of aerogels [56]. Tortuosity was shown to be on the order of 1 to 3 for highly porous aerogels [11].

The binary diffusion coefficient is a function of CO_2_ mole fraction [46]. Composition dependence of binary diffusion coefficient of pore liquid and CO_2_ can be predicted using an empirical correlation given by Equation (6) which was proposed by Vignes et al. for concentrated liquid mixtures where $D_{12}^{\infty }$ is binary diffusion coefficient of CO_2_ in the pore liquid at infinite dilution and $D_{21}^{\infty }$ is binary diffusion coefficient of the pore liquid in scCO_2_ at infinite dilution, *x*_1_ is mole fraction of CO_2_, and *α* is a thermodynamic correction factor assumed to be 1 based on the ideal solution approximation for the binary solution in the pores [57]. It is generally agreed that Vignes expression gives excellent agreement with experimental results in the case of ideal solutions [57,58].

(6) $$D_{12}={\left(D_{12}^{\infty }\right)}^{x_{2}}{\left(D_{21}^{\infty }\right)}^{x_{1}}\alpha$$

**Figure 8:**
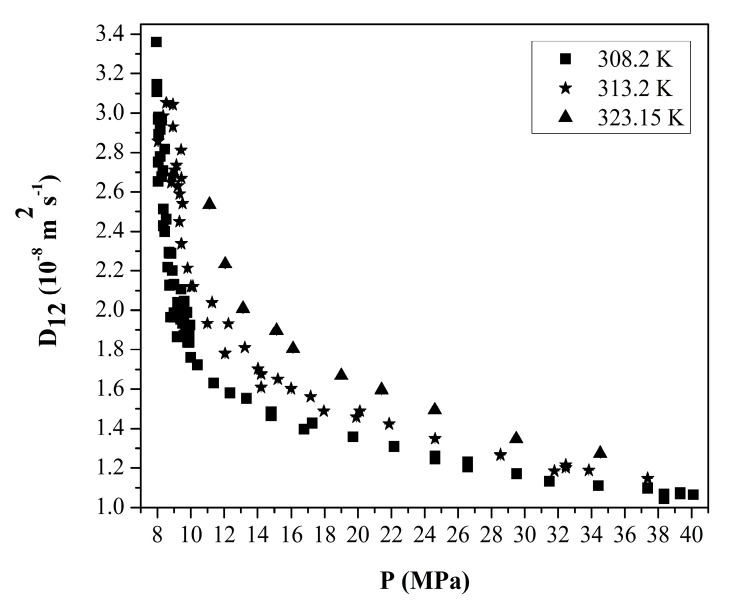
Binary diffusion coefficients of acetone in scCO_2_ at infinite dilution obtained by curve fitting in the time domain (FTD) method vs. pressure at three different temperatures. Adopted with permissions from [59,60].

Since the pores of the wet gel are filled with organic solvents, commonly ethanol and acetone, their binary diffusion coefficients in scCO_2_ at infinite dilution are needed to utilize Equation (6). Many studies on the measurements of the infinite-dilution diffusion coefficients for binary systems of organic solvents and CO_2_ were reported in the literature, and most of them employed the Taylor dispersion technique. In this measurement technique, a small amount of a solute is injected into a cylindrical diffusion column through which scCO_2_ is flowing in fully developed laminar flow. Tracer concentration profile as a function of time is obtained at the column exit using a detector. The analysis of the profiles enables one to extract the diffusion coefficient. Figure 8 gives the binary diffusion coefficient of acetone in scCO_2_ at infinite dilution as a function of pressure at 308 and 323 K measured by Funazukuri et al. [59,60].

**Figure 9:**
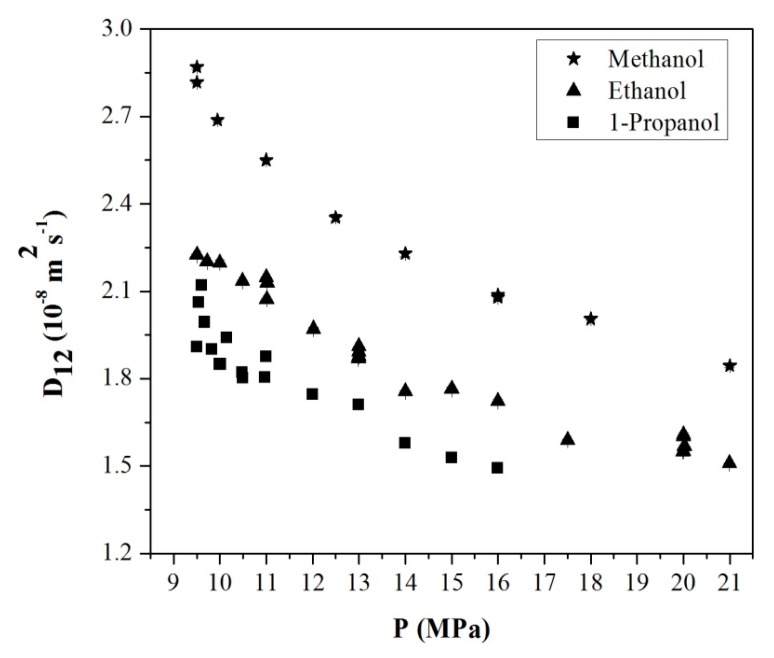
Binary diffusion coefficient of various organic solvents in scCO_2_ at infinite dilution at 313.15 K as a function of pressure. Adopted with permission from [61]. Copyright (2017) American Chemical Society.

Kong et al. also measured the infinite dilution diffusion coefficients of a variety of polar compounds such as mono-alcohols and at 313.2 K at pressures higher than 9.5 MPa by using a technique called chromatographic impulse response (CIR) to diminish errors in the Taylor dispersion method [61]. In this technique, a polymer coated capillary tube is used as a diffusion column and a model is used to describe partitioning of a solute species between the polymer phase and supercritical phase. Measured binary diffusion coefficients of various organic solvents in scCO_2_ at infinite dilution at 313.15 K measured using CIR are given in Figure 9 [61].

Infinite dilution binary diffusion coefficients of organic solvents in scCO_2_ may also be estimated by several available empirical correlations. There have been a large number of studies in the literature for prediction of diffusion coefficients of compounds in supercritical fluids in binary systems and correlations have been developed for various solutes. The predictive equations of binary diffusion coefficients in supercritical fluids at infinite dilution are divided into two categories as based on Stokes-Einstein theory and those inspired by the rough hard sphere model [62]. The Stokes-Einstein theory assumes that limiting binary diffusivities are a function of absolute temperature, solvent viscosity and molecular diameter of solute [63,64,65]. The rough hard sphere theory is based on Enskog-Thorne’s hard-sphere model [62,66]. The following empirical equations in Table 2 from Equations (7) to (10) are widely used to effectively predict diffusion coefficients of solutes in supercritical fluids at infinite dilution.

**Figure 10:**
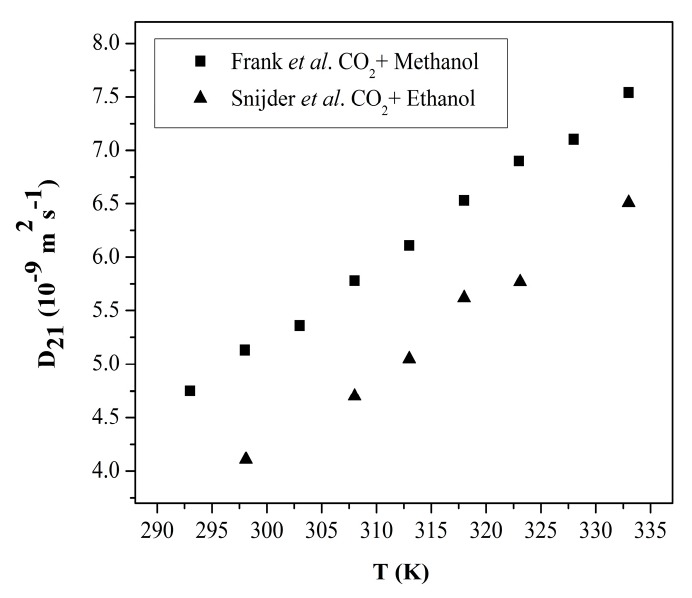
Diffusion coefficients of CO_2_ in methanol and ethanol at infinite dilution as a function of temperature. Adopted with permission from [64,71]. Copyright (2017) American Chemical Society.

The other term in the Vignes correlation is the infinite dilution diffusion coefficient of CO_2_ in the organic solvent. These can be calculated using the correlations developed for diffusion of gases in liquids assuming that it is almost pressure-independent since the solvent density does not change appreciably when pressure is raised from 1 atm to pressures employed in supercritical drying. There are also some available experimental data for infinite dilution binary diffusion coefficients of CO_2_ in various organic liquids at varying temperatures. These measured diffusion coefficients of CO_2_ in various organic solvents at infinite dilution are provided in Figure 10 and Table 3.

Binary diffusion coefficients of CO_2_ in some organic solvents were also correlated. Snijder et al. provided a correlation for diffusion coefficient of CO_2_ in ethanol for the range of 298 to 333 K given by [71]

(11) $$D_{21}\left(m^{2}s^{-1}\right)=336.5\times 10^{-9}exp\left(\frac{-1314.7}{T/K}\right)$$

Frank et al. also correlated diffusion coefficient of CO_2_ in methanol as follows [64]

(12) $$D_{21}\left(m^{2}s^{-1}\right)=2.22\times 10^{-7}exp\left(\frac{-9340}{RT}\right)$$

Some of the estimates in the literature for effective diffusivities in aerogels are given in Table 4.

#### 3.2.2. Convective Mass Transfer

The mass flux from the gel surface towards the flowing stream and the mass flux due to convection in the flowing stream are described by Equations (13) and (14), respectively.

(13) $$N_{a,s}=k_{x}\left(C_{s}-C_{v}\right)$$

(14) $$N_{a,z}=C_{v}v_{z}$$

where $\;N_{a,s}$ is convective mass flux of pore liquid from the gel surface into the flowing stream, $N_{a,z}$ is convective mass flux of pore liquid in the flowing stream, $k_{x}$ is an external mass transfer coefficient, *C_s_* is concentration of the pore liquid on the gel surface, $C_{v}$ is the average concentration of pore liquid in the flowing stream, $v_{z}$ is velocity of the scCO_2_.

Mass transfer coefficient values can be calculated using Sherwood number (Sh) correlations as a function of Schmidt number (Sc) and Reynolds number (Re) in the literature expressed in the form of a general equation, as Sh = cRe^a^Sc^b^ [74]. However, it is important to point out that no published correlations are available for prediction of overall mass transfer coefficient for the supercritical extraction of liquid from a porous material. Among the various correlations in the literature, a couple of Sherwood number correlations could be applicable to such a system. Wakao and Kaguei proposed a correlation for particle-to-fluid forced convection mass transfer in packed beds. This correlation was also considered appropriate for the evaluation of mass transfer in a packed bed with a supercritical fluid [75]. Tan et al. [76] measured extraction rates of β-naphthol in supercritical CO_2_ with three sizes of soil particles in a packed-bed reactor and developed a mass transfer correlation. Puiggené et al. [75] developed a correlation for evaporation of 1,2-dichlorobenzene deposited on a shallow bed of non-porous and non-adsorbing glass beads into a scCO_2_ stream in a packed bed. Stüber et al. [74] correlated their mass transfer data for estimation of external, particle-to-fluid mass transfer coefficients of 1,2-dichlorobenzene and toluene in scCO_2_ in sintered metallic pellets as inert solid packings. These mass transfer coefficient correlations are tabulated in Table 5. In a study on mass transfer in supercritical drying of cylindrical silica alcogel monoliths, Özbakır et al. [5] regressed the mass transfer coefficient by fitting the concentration versus time data to model equations. The regressed values of the mass transfer coefficient for the alcogel extraction were in the same order of magnitude as values calculated from the correlations. It was shown that the mass transfer coefficients agreed slightly better with the correlations proposed by Wakao and Kaguei [77] and Tan et al. [76] than the correlations by Puiggené [75] and Stüber [74]. The values calculated by Wakao and Kaguei and Tan et al. correlations were lower than the regressed values approximately by a factor of three. 

#### 3.2.3. Spillage by Volume Expansion

**Figure 11:**
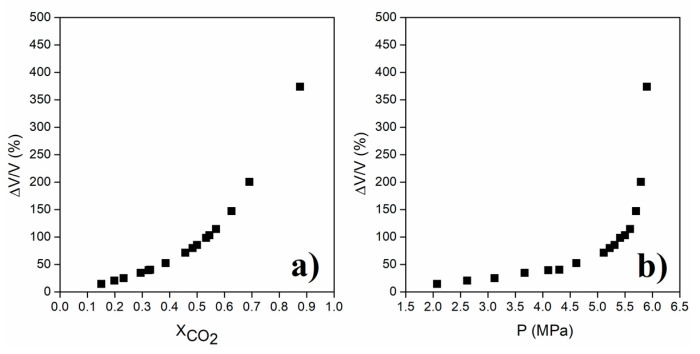
Relative volume expansion of ethanol vs. liquid mole fraction of carbon dioxide (**a**) and pressure (**b**) at 298.15 K. Plots are regenerated using the data available at [79].

Another important phenomenon which is speculated to be present along with diffusion and convection is the spillage of the solvent from the pores due to volume expansion by dissolution of CO_2_ into pore liquid. The concept of expanded liquids is usually encountered in gas-antisolvent process in which dissolved gas decreases the solubility of the solute in the solvent resulting in precipitation of the solids [79,80]. Solvation power of organic solvents to dissolve CO_2_ was found to increase with pressure, leading to substantial increase in percent volume change as in Figure 11 [79]. Relative volume expansion of a system in the presence of CO_2_ can be calculated using Equation (15) [80] as follows

(15) $$\frac{\Delta V}{V}=\frac{\rho_{2}\left(T,P_{0}\right)}{\rho_{L}\left(T,P,x_{1}\right)}\left(\frac{x_{1}}{1-x_{1}}\frac{M_{1}}{M_{2}}+1\right)-1$$

where *ΔV*/*V* is relative volume expansion, *T* is temperature, *P* is pressure, $P_{0}$ is the ambient pressure, $\rho_{L}$ and $\rho_{2}$ are density of liquid mixture and the solvent, respectively; $x_{1}$ is the mole fraction of the solvent, $M_{1}$ and $M_{2}$ are molecular weights of carbon dioxide and solvent, respectively.

**Figure 12:**
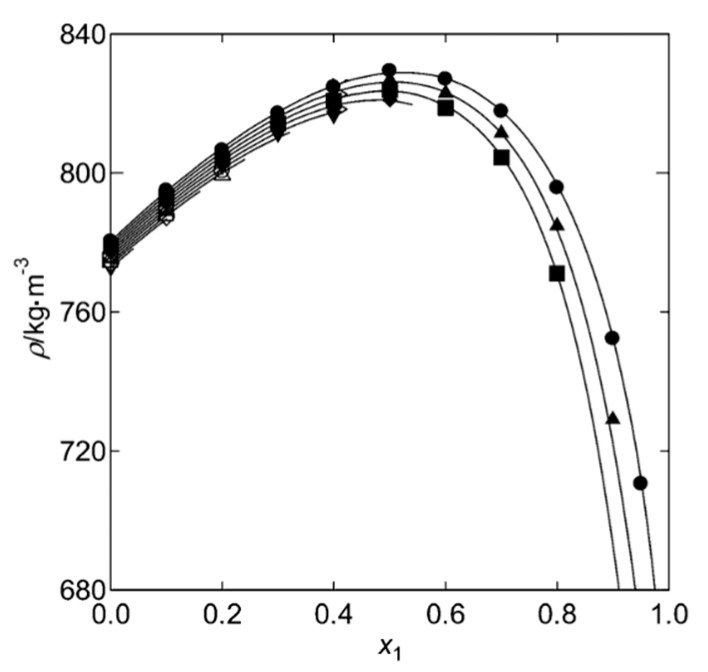
Densities of CO_2_ + ethanol mixture with respect to mole fraction of CO_2_ at 313.15 K: ●, 10 MPa; ▲, 9 MPa; ■, 8 MPa; ♦, 7 MPa; ▼, 6 MPa; ○, 5 MPa; Δ, 4 MPa; □, 3 MPa; ◊, 2 MPa; ▽, 1 MPa. Adapted with permission from [81]. Copyright 2017 American Chemical Society.

**Figure 13:**
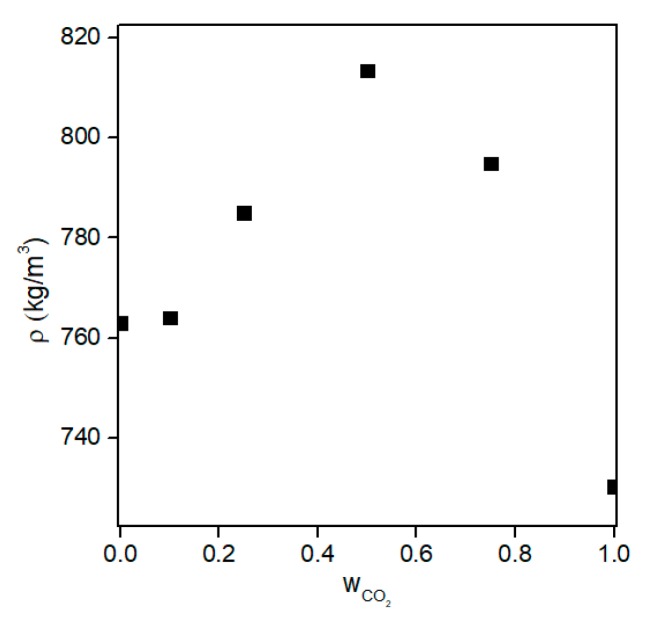
Density of acetone-CO_2_ mixture with respect to CO_2_ weight fraction at ~325 K and ~14 MPa. Data is taken from [82]. Copyright 2017 American Chemical Society.

Equation (15) shows that percent volume expansion depends on the density of the solvent-CO_2_ mixture in the pores and mole fraction of CO_2_ in the same mixture. At the initial stage of supercritical drying, mole fraction of the solvent inside the pores is accepted as 1 since there is only solvent in the pores. Then, CO_2_ diffuses into pores whereas the solvent diffuses in the reverse direction, resulting in an increase of the mole fraction of CO_2_ in the solvent. Consequently, density of the solvent-CO_2_ mixture in the pores varies during drying. Figure 12 and Figure 13 show how density varies with composition for ethanol-CO_2_ and acetone-CO_2_ systems for temperatures and pressures commonly employed in supercritical drying. Maximum percent density changes of the systems between the start of the drying until the point where mole fraction of CO_2_ is 0.95 can be calculated from the plots as 9% and 4% for ethanol and acetone mixtures, respectively from the data given in Figure 12 and Figure 13. Since the percent density change is small, the main parameter determining the relative volume expansion would be the mole fraction of CO_2_. Therefore, the amount of volume expansion would most likely be determined by the mole fraction of CO_2_ in the pores which depends on how fast CO_2_ diffuses into the pores. Since volume of the pores can be considered as constant during drying, volume expansion would eventually lead to spillage of the pore filling liquid to CO_2_ stream. During volume expansion and consequent pressure increase of liquid in the pores, resistances due to surface tension and friction by pore walls are overcome so that there would be a flow and spillage of the expanded liquid. Therefore, both operating conditions and textural properties of gel would affect this transport mechanism.

Some researchers tried to incorporate the transport mechanism via spillage of the solvent by volume expansion into a theoretical model of supercritical drying of ethanol/silica gel system [29,46]. Mixture molar volumes and percent change in the solvent volume were calculated using the Peng-Robinson equation of state with correction for pressure in the pores by Kelvin equation. However, the studies are lacking experimental justification to show the presence of spillage phenomenon.

#### 3.2.4. Axial Dispersion

Axial dispersion is a phenomenon in which the concentration of the solvent in flowing CO_2_ stream changes in the flow direction due to diffusion in the axial direction and non-uniform velocity profiles. In the case of supercritical drying of packed bed of gel particles, axial dispersion of the solvent in the flowing CO_2_ stream should also be considered. A criterion was proposed by Han et al. as follows [83]:

(16) $$\frac{L}{d_{p}}\frac{1}{P_{e}}\frac{1-\in_{b}}{\in_{b}}\geq 0.3$$

where *L* is bed length, *d_p_* is particle diameter, *Pe* is Peclet number, and $\in_{b}$ is bed void fraction. 

If this criterion is satisfied, axial dispersion coefficients would not change through bed length. *Pe* is a ratio of convective to disperse flow in the axial direction which is usually correlated in terms of Reynolds and Schmidt numbers. Axial dispersion can be avoided by ensuring very high Peclet numbers, high interstitial solvent velocity, small particle diameter, and longer beds [84]. It is also generally accepted that if the ratio of bed length to particle diameter is larger than 50, the effect of bed length on axial dispersion can be neglected [85]. However, this phenomenon should be taken into account by using an effective axial dispersion coefficient if the conditions above are not satisfied. Effective dispersion coefficients can be estimated by using correlations as a function of Reynolds and Schmidt numbers developed for supercritical extraction process [84]. On the other hand, there is no literature on experimental and theoretical evaluation of mass transfer by axial dispersion during supercritical drying of gels.

### 3.3. Models for Kinetics of Supercritical Drying

Aerogel and vessel geometry are important for derived model equations to be sufficiently well representative of the drying process inside the vessel and the boundary conditions are well-defined in the system. It has been experimentally challenging to set up such drying experiments. Two studies in the literature have overcome this problem using a cylindrical gel with a CO_2_ phase flowing around it [5,31]. Model equations representing drying of the cylindrical gel phase and in the flowing scCO_2_ phase in the tubular extraction vessel are developed from a mass balance over differential volume elements in the aerogel phase and the flowing scCO_2_ phase. Mass transfer occurring in the gel phase and flowing scCO_2_ phase are described by two coupled partial differential equations. For the gel phase, two dimensional diffusion of the pore liquid through axial direction and radial direction in the gel are modelled based on Fick’s second law. For the flowing stream, mass transfer of the pore liquid by convection from the gel surface to the continuous scCO_2_ phase and mass transfer by convection in the axial direction in the tubular vessel are considered.

The partial differential equation obtained from the mass balance in a differential volume element in a cylindrical gel rod is given by

(17) $$\frac{1}{R}\frac{\partial }{\partial r}\left(R\;D_{e}\frac{\partial C_{a}}{\partial r}\right)+\frac{1}{R}\left(D_{e}\frac{\partial C_{a}}{\partial r}\right)+\frac{\partial }{\partial z}\left(D_{e}\frac{\partial C_{a}}{\partial z}\right)=\frac{\partial C_{a}}{\partial t}$$

where *C_a_* is concentration of the pore liquid in the gel, (kmol/m^3^); $D_{e}$ is effective diffusion coefficient, (m^2^/s); R is radius of the gel (m); *t* is time (s); *z* and *r* is the direction that diffusion occurs in the gel.

The partial differential equation obtained from a mass balance in a differential volume element outside of the gel in the fluid phase is given by

(18) $$\frac{2Rk_{x}\left(C_{s}-C_{v}\right)}{\left({R_{v}}^{2}-R^{2}\right)}-v_{z}\frac{\partial C_{v}}{\partial z}=\frac{\partial C_{v}}{\partial t}$$

where $k_{x}\;$ is an external mass transfer coefficient (m/s); *R_v_* is inner radius of the vessel (m); $v_{z}$ is velocity of scCO_2_ (m/s).

The left hand side of Equation (18) accounts for convective mass transfer of the pore liquid, the first term represents the mass transfer taking place between the gel surface and the flowing stream of scCO_2_ and the second term denotes the mass transfer of the pore liquid by convection in the *z* direction in the vessel. The right hand side of the equation represents the accumulation of the pore liquid in the flowing stream. In Equation (18), $v_{z}$ is assumed to be constant and is given by

(19) $$v_{z}=\frac{Q}{A_{c}}$$

(20) $$Q=\frac{ṁ}{\rho }$$

where Q is the volumetric flow rate of scCO_2_ (m^3^/s); $A_{c}$ is cross sectional area of the annulus between the vessel wall and aerogel surface (m^2^); ṁ is mass flow rate of the scCO_2_ (kg/s); ρ is density of scCO_2_ (kg/m^3^); ṁ is constant in the system, and since change in ρ is negligible, Q is assumed to be constant in the system. Since $A_{c}$ and Q are constant in the system, $v_{z}$ is constant.

The initial and boundary conditions are given by:

Initial conditions:

(21) $$t=0\;C_{a}\left(r,z,0\right)=C_{i}$$

(22) $$t=0\;C_{v}\left(z,0\right)=0$$

Boundary conditions:

(23) $$t>0\;\frac{\partial C_{a}}{\partial r}=0\;\mathrm{at}\;r=0$$

(24) $$t>0\;N_{a,z}=\;N_{a,s}\;at\;z=0$$

(25) $$N_{a,z}=-D_{e}\frac{\partial C_{a}}{\partial z},\;D_{e}\frac{\partial C_{a}}{\partial z}{|}_{surface}=k_{x}\left(C_{v}{|}_{z=0}-C_{s}{|}_{z=0}\right)\;\mathrm{at}\;z=0$$

(26) $$t>0\;\frac{\partial C_{a}}{\partial z}=0\;\mathrm{at}\;z=L$$

(27) $$t>0\;N_{a,r}=N_{a,s}\;\mathrm{at}\;r_{a}=r$$

(28) $$N_{a,r}=-D_{e}\frac{\partial C_{a}}{\partial r},\;D_{e}\frac{\partial C_{a}}{\partial r}{|}_{surface}=k_{x}\left(C_{v}-C_{s}\right)\;\mathrm{at}\;r_{a}=r$$

where $C_{i}$ is initial concentration of pore liquid, *L* is the length of the gel, $r_{a}$ is radius of the gel, $k_{x}$ is mass transfer coefficient, $N_{a,s}$ is convective mass flux of pore liquid from the gel surface into the flowing stream, $N_{a,z}$ is diffusive flux of pore liquid within the gel in the *z* direction, and $N_{a,r}$ is diffusive flux of pore liquid within the gel in the r direction.

The initial condition in Equation (21) indicates that the concentration of pore liquid is initially uniform throughout the gel volume with a concentration of *C_i_* which is the concentration of the pore liquid at the initial conditions. Equation (22) demonstrates that the flowing stream of scCO_2_ is free from the pore liquid at the beginning of the process (*t* = 0). Mass flux of the pore liquid by diffusion at the center and at the bottom of the gel is assumed to be zero, given in Equations (23) and (26). Mass flux of the pore liquid from the gel surface to the flowing stream is described by convective mass transfer. Equations (27) and (28) show that the convective mass flux of the pore liquid at the gel surface is equal to diffusive mass flux of the pore liquid at the interface between the gel and the flowing stream. A similar model was also used by Griffin et al. A composition dependent diffusivity was used with no external mass transfer coefficient, however, they accounted for the developing velocity profile.

Similar equations can be written for the case of superficial drying of spherical gel particles in a packed bed. Partial differential equation governing mass transfer of the solvent inside a spherical gel is given by

(29) $$\frac{\partial }{\partial r}\left(D_{e}\frac{\partial C_{a}}{\partial r}\right)+\frac{2}{r}D_{e}\frac{\partial C_{a}}{\partial r}=\;\frac{\partial C_{a}}{\partial t}$$

The mass balance of the solvent in the flowing carbon dioxide stream along in the flow direction yields

(30) $$-v_{z}\frac{\partial C_{v}}{\partial z}+\frac{3k\left(1-\varepsilon_{b}\right)}{R\varepsilon_{b}}\left(C_{s}-C_{v}\right)=\frac{\partial C_{v}}{\partial t}$$

where *r* is the radial direction, *R* is the particle radius, *k* is the mass transfer coefficient, and *ε_b_* is the void fraction of the packed bed.

The initial and boundary conditions are given by

Initial conditions:

(31) $$t=0\;C_{a}\left(r,0\right)=C_{i}\;\mathrm{for}\;\mathrm{all}\;z$$

(32) $$t=0\;C_{v}\left(z,0\right)=0\;\mathrm{for}\;\mathrm{all}\;z$$

Boundary conditions:

(33) $$t>0\;\frac{\partial C_{a}}{\partial r}=0\;\mathrm{at}\;r=0$$

(34) $$t>0\;\frac{\partial C_{v}}{\partial z}=0\;\mathrm{at}\;z=0\;\mathrm{and}\;z=L$$

(35) $$t>0\;N_{a,r}=-D_{e}\frac{\partial C_{a}}{\partial r},\;D_{e}\frac{\partial C_{a}}{\partial r}{|}_{surface}=k\left(C_{v}-C_{s}\right)\;\mathrm{at}\;r=R\;\mathrm{for}\;\mathrm{all}\;z$$

Temperature and pressure are significant operating parameters affecting the kinetics of the drying process due to large changes of density and viscosity with these parameters.

### 3.4. Review of Studies on Drying Kinetics

There are a few studies in the literature on modeling of supercritical drying of alcogels with scCO_2_. The models evolved over time from 1D mass transfer models involving constant diffusion coefficients to 2D mass transfer models incorporating composition dependent diffusion coefficients. In most of the studies, model results were further compared with experimental data for validation. On the other hand, some research was focused only on mathematical modeling whereas some others involved only experimental work to better understand supercritical drying kinetics.

**Figure 14:**
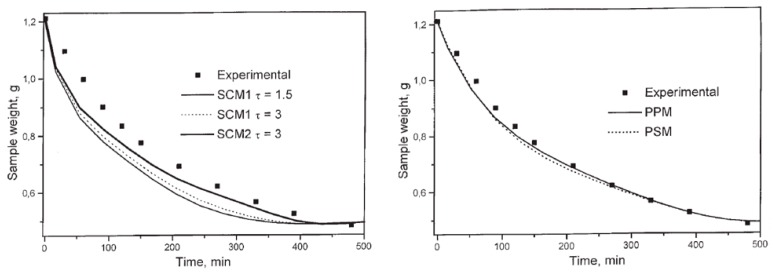
Comparison of experimental data with model results (SCM: shrinking core model; PPM: parallel pore model; PSM: pore in series model). Adopted with permission from [86].

As an example of 1D mass transfer models, the study of Orlovic et al., on the drying of alumina/silica alcogel the pores of which were filled with 1-butanol can be given [86]. The developed model was represented as an unsteady one-dimensional diffusion of solvent through pores of the gel. Drying of the alcogels was also experimentally monitored by the change of the weight of wet gel with time. The researchers tested four models which were shrinking core model 1 and 2, parallel pore model, and pore in series model. In shrinking core model 1, they neglected Knudsen diffusivity and used an average overall diffusivity. Whereas in shrinking core model 2, they included Knudsen diffusivity. In those models, the use of a single diffusivity value for the whole aerogel sample failed to predict the drying behavior accurately. However, the parallel pore model and the pore in series model which were developed on the basis of measured porous structure of the aerogel, involved the use of different diffusivity values for each pore size and consequently resulted in better agreement with experimental data as seen in Figure 14.

**Figure 15:**
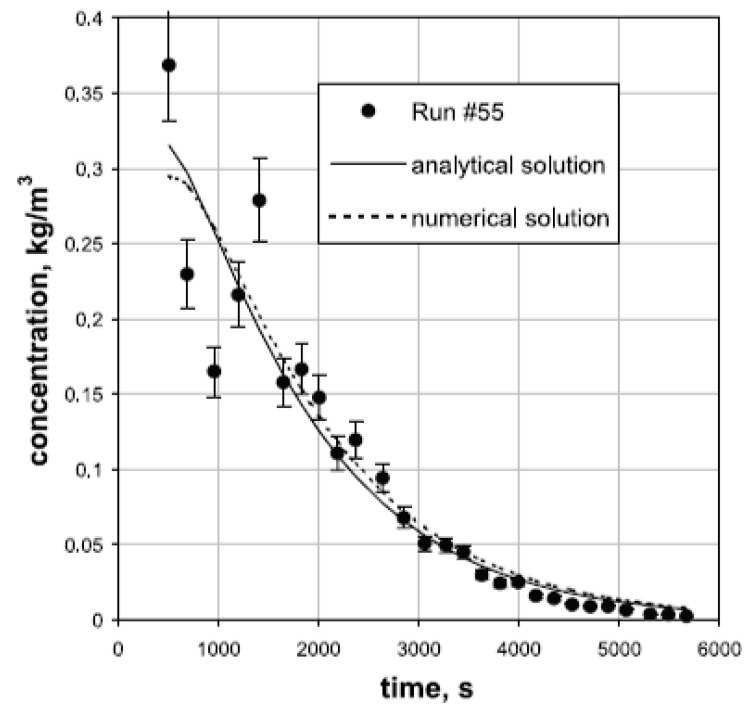
Comparison of the experimental data to the model results (both analytical and numerical solutions). Adopted with permission from [38].

In another study, a one-dimensional, transient, and analytical diffusion model was developed with the assumption of zero ethanol concentration along the outer diameter of the gel [27]. Further study by Wawrzyniak et al. involved investigation of the mathematical modeling of the supercritical drying again using a model based on the solution of Fick’s equation in an infinite cylinder, thereby only accounting for the effect of diffusion [38]. The equations were solved both analytically and numerically as shown in Figure 15. Assumption of zero ethanol concentration outside of the alcogel for the analytical solution resulted in greater deviations from the experimental results compared to the numerical solution.

Another study involving the use of an analytical solution to describe supercritical drying kinetics was carried out by Masmoudi et al. [39]. Similar assumptions were used to obtain an analytical solution of Fick’s equation for plane sheets considering instantaneous evacuation of the solvent on the gel surface. There was a deviation of model results from the experimental data which was attributed to assumptions such as the consideration of zero ethanol concentration at the gel surface, the use of constant diffusion coefficient instead of a composition dependent one and the use of constant flow rate.

**Figure 16:**
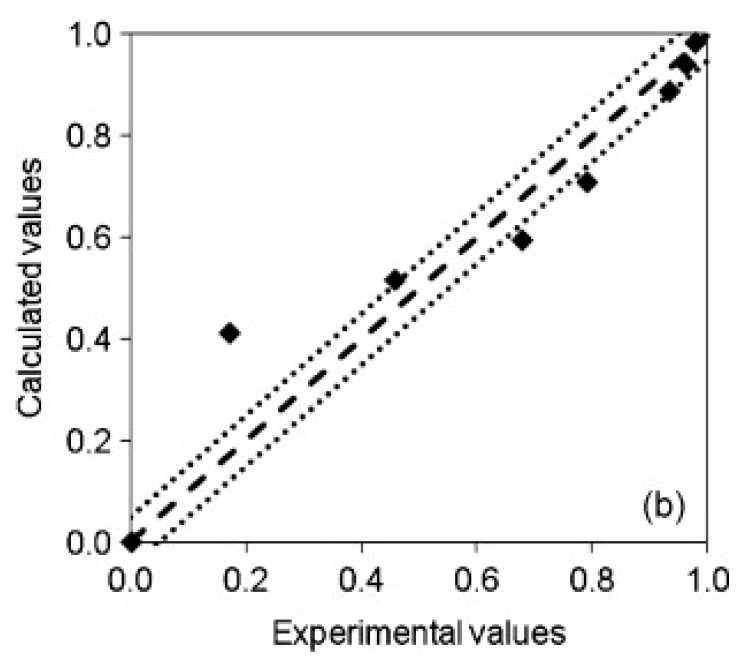
Normalized parity plot of experimental values of ethanol extracted by supercritical drying of a starch aerogel. Adopted with permission from [33].

In the study of Garcia-Gonzalez et al., the amount of ethanol removed as a function of time was calculated using again the analytical solution of a model based on Fick’s second law but this time in cylindrical coordinates [33]. It was assumed that Fickian diffusion was the single mass transfer mechanism for the modeling of ethanol removal from silica alcogels. As a consequence, the model failed to predict the initial drying stage as seen in Figure 16 where convective mass transfer was also significant. However, the later stages of the drying were accurately predicted with the same model indicating that the last stage of the supercritical drying should be diffusion controlled.

To be more realistic in terms of mass transfer in supercritical drying, 2D mass transfer models were also proposed. A purely diffusive mass transfer model incorporating a composition dependent effective diffusivity was proposed by Griffin et al. [31]. An aerogel with annular geometry and laminar internal flow of scCO_2_ were considered in this model. Mass transfer was based on molecular diffusion. The use of a composition dependent diffusion coefficient rather than a constant one led to a good agreement between the model and experimental data.

**Figure 17:**
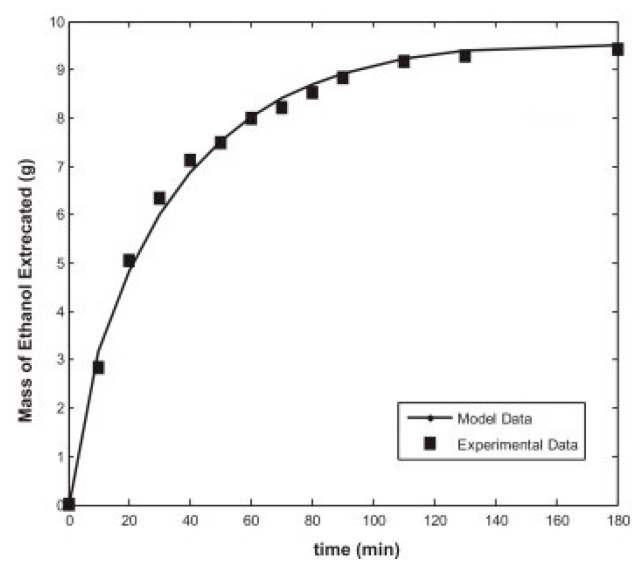
Comparison of experimental extraction profile of a silica alcogel to model data at 100 bar, 40 °C [5].

Subsequently, a more comprehensive 2D model was proposed by Ozbakır and Erkey [5]. The model treated the alcogel phase and flowing scCO_2_ phase separately. 2D (axial and radial) diffusive model based on Fick’s second law was considered for the transfer of ethanol from alcogel to flowing stream. On the other hand, transfer mechanisms in the flowing scCO_2_ stream were convective mass transfer of ethanol from alcogel surface to bulk CO_2_ and convection in the axial direction. There was a good agreement between the proposed model results and experimental percent removal data as a function of time as shown in Figure 17.

Mukhopadhyay and Rao developed a two way mass transfer model for the supercritical drying of silica alcogel [46]. They used a parallel-pore configuration where each pore had one end closed and the other end open to scCO_2_ stream. The model mechanism considered the initial diffusion of scCO_2_ to ethanol filled pores. High dissolution CO_2_ would then lead to spillage of ethanol from pores due to volume expansion. Fick’s second law of diffusion was assumed to be valid for dissolution of scCO_2_, taking into account both molecular and Knudsen diffusion with composition dependent binary liquid phase diffusivity of ethanol-CO_2_. The volume expansion in ethanol was described by Peng-Robinson equation of state. Lastly, convective transport of ethanol away from the open-end of the pore to the flowing CO_2_ stream was taken into account using a mass transfer coefficient predicted by a Sherwood number correlation.

Recently, Lebedev et al. developed a mathematical model based on continuum mechanics [29]. Only the diffusion transport mechanism was considered in the modeling of mass transfer of ethanol from pores of silica alcogel. Simulations were carried out using the model to investigate the effects of process parameters such as flow rate, gel thickness and dryer geometries. Obtained results provided insights on the mass transfer in the free volume of the reactor, on the gel surface, and also inside the gel. Based on those results, it was seen that the removal of solvent from the free volume occurred mainly in the first two hours where mass transfer from inside the gel was very slow. This was followed by an increased rate of mass transfer by diffusion from inside the gel with decreasing solvent concentration at the surface of the gel.

Another approach was adopted by Novak and Knez [37]. They observed non-transparent and cracked samples at the end of drying. The non-transparent area was thought to be due to insufficient drying, whereas crack formation was related to the transformation of unextracted methanol to vapor phase during depressurization, leading to subsequent evaporation of the liquid-gas phase, damaging the sample. It was suggested that the determination of the binary diffusion coefficient using the data on how the width of the non-transparent area changes as a function of time would enable prediction of drying time for a crack free and transparent aerogel. Rogacki and Wawrzyniak also reached the same conclusion, stating that the size and the shape of a damaged, non-transparent zone can be predicted as a function of time for simple geometries [35]. Thereby, comparison of the model data with experimental results might enable the identification of the kinetic parameters.

**Figure 18:**
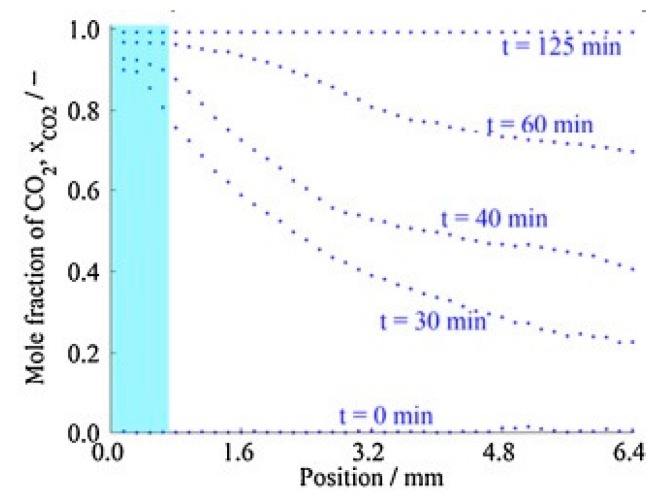
Representative composition profiles xCO_2_ at different times (light blue color indicate the region out of the gel) [34].

Another experimental study on supercritical drying was carried out by Quino et al. where one-dimensional Raman spectroscopy was used to provide some insights into the mass transport processes that are involved during supercritical drying of a silica gel [34]. They have visualized in situ temporally and spatially resolved composition and concentration fields developing inside a silica gel monolith during drying as shown in Figure 18. The evolution of concentration fields showed that ethanol was continuously transported out of the gel.

Sans-Moralez et al. investigated the effect of size on mass transfer mechanisms during supercritical drying of silica aerogels using a view cell to better understand drying kinetics experimentally [30]. Based on the findings, earlier stages of drying were dominated by convection, whereas following stages were controlled by the diffusion of the solvent from the pores. Moreover, analysis of video captures allowed researchers to optimize the drying time.

**Figure 19:**
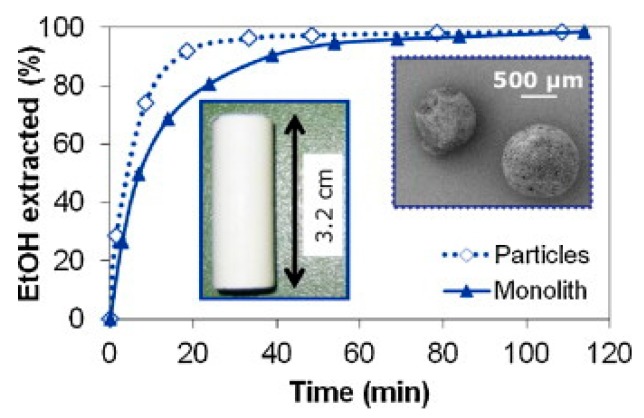
Comparison of supercritical drying profiles of starch aerogel particles and monoliths. Adopted with permission from [32]. Picture on the left: monolith, picture on the right: particles.

The kinetics of supercritical drying of particles were first studied by Gonzalez and Smirnova who experimentally compared the drying kinetics of starch aerogel particles and monoliths [32]. Longer drying times for monoliths compared to particles was related to diffusion based mass transfer. Shorter diffusion paths in aerogel particles had resulted in faster drying. Figure 19 shows the percent ethanol extraction profile for monolithic and particle gels.

Although kinetic models were based upon different assumptions and mass transfer mechanisms, the investigated parameters were almost the same. Mostly, the effect of operating conditions such as temperature, pressure, and flow rate with the effect of some gel parameters such as gel thickness were studied in the literature.

Bommel and de Han investigated drying of monolithic silica aerogel sheets. Modification of drying conditions (from 85 bar, 35 °C to 140 bar, 70 °C) had only a minor effect on the drying [36]. However, the findings of the study of Orlovic et al. indicated that increasing the temperature affected the drying time considerably. As elevated temperatures led to an increased solubility of the solvent used (1-butanol), drying times were significantly reduced [86]. It was shown that changing operating conditions such as temperature and pressure affected the diffusion coefficient which thereby affected the drying time. Mukhopadhyay and Rao tested only two similar temperatures in their model which were 308 and 313 K [46]. However, they also reached the same conclusion, stating that the lower temperatures led to longer drying times due to lower convective mass flux of ethanol and lower diffusion coefficient of ethanol in carbon dioxide. Ozbakır and Erkey showed that increasing the diffusion coefficient 10-fold had significant effect on the drying time [5]. Duration of drying with the largest diffusion coefficient was almost 2 h, whereas the reduction of ethanol concentration within the pores was around 40% with the use of smaller diffusion coefficients in the same time interval.

Another commonly investigated parameter was the effect of the gel thickness on the drying time. As expected, drying time was shown to increase with increasing gel thickness. Bommel and de Han used a dimensionless mass transfer Fourier number assuming that a properly dried gel should have the same Fourier number and diffusion coefficient at constant operating conditions to characterize the drying process [36]. It was concluded that the time required for drying should be based on the plate thickness or the diameter.

Griffin et al. also concluded that the required drying time might be scaled with thickness squared based on simple scaling arguments [31]. However, the assumption of zero solvent concentration at the gel surface led to some errors due to deviation from the actual situation. A similar effect of the gel thickness on the drying time was also observed in other studies indicating that reducing the gel thickness significantly decreased the required drying time [5,46,86]. Flow rate of supercritical carbon dioxide was another important parameter which might affect the drying time. Based on the kinetic model of Mukhopadhyay and Rao, increasing flow rate decreased the drying time due to an increase in the convective flux of ethanol from the pores [46]. A similar conclusion was reached by Lebedev et al., showing that increased flow rates reduced the drying times [29]. On the other hand, the analysis carried out by Griffin et al. indicated that, beyond the range where mass transfer driving force might be decreased by excessive ethanol build up in flowing CO_2_ stream, the process is relatively independent on the mass flow rate [31]. Only at the beginning of the drying, the extraction rate was increased by increasing mass flow rate. A similar conclusion was also reached in the study of Ozbakır and Erkey who demonstrated that simulated values of percent ethanol removal at different flow rates were nearly identical [5].

**Figure 20:**
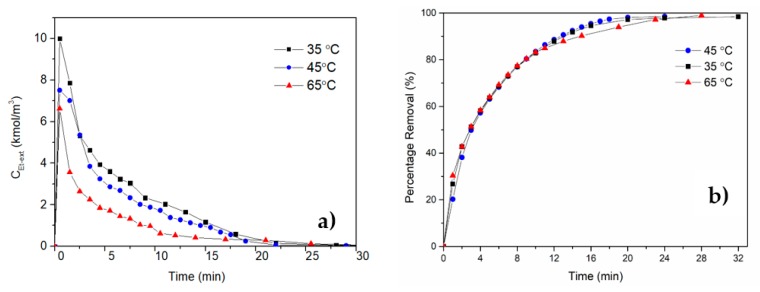
Ethanol exit concentration change (**a**) and percent removal (**b**) with time for different temperatures for supercritical drying of calcium alginate beads.

Previous studies on drying kinetics discussed above show that supercritical of drying of a bed of gel particles was not studied in detail. We recently studied the effect of operating conditions on drying kinetics in a fixed bed of calcium alginate alcogels in the form of spherical beads with a particle size of 0.4 cm. Experiments were carried out in three different temperatures at 10 MPa with an exit CO_2_ gas flow rate of 2 L/min to investigate the effect of temperature on concentration profiles of ethanol at the exit of the extraction vessel. Results in Figure 20 indicate that, although temperature changes the exit ethanol concentration profiles mainly due to change in density, drying time is not affected considerably.

## 4. Conclusions and Future Research Directions

Based on the studies mentioned above, current understanding of the supercritical drying process is mostly based on studies on silica alcogel based systems where ethanol is used as the solvent. Earlier models used constant effective diffusion coefficients, however, the importance of using a composition dependent effective diffusion coefficient and a mass transfer coefficient is emphasized in recent research. Studies indicated that the effect of convection should not be omitted but must be included along with diffusion. Since the drying is a very complex process composed of different transport phenomena, future research efforts should focus on including as many different phenomena as possible in the models. Therefore, solvent spillage by volume expansion and axial dispersion should be incorporated into models reflecting the underlying physics. On the other hand, preparation of aerogels in the form of particles rather than monoliths is attracting increased attention along with organic and hybrid aerogels. Therefore, both experimental and modeling studies are needed to understand drying kinetics of aerogel particles. Future research efforts should also focus on gel-solvent pairs other than silica gel-ethanol.

In conclusion, current literature on kinetics of supercritical drying of gels indicate that we do not fully understand the supercritical drying process. Research is needed on the effects of operating conditions and gel properties on kinetics. Consequently, kinetics of supercritical drying of gels is expected to attract increasing attention from the aerogel community in the near future.

## Figures and Tables

**Table 1:** Values of capillary tension for different solvents and pore sizes calculated for *T* = 20 °C and δ = 1 nm using Equation (1).

Solvents	*γ_LV_* * in 10^−3^ Nm^−1^	*P_c_* in Bar for *d* = 5 nm	*P_c_* in Bar for *d* = 50 nm	*P_c_* in Bar for *d* = 500 nm
Water	73.1 **	974	60.9	5.9
Ethanol	22.8	303	19.0	1.8
Methanol	22.6	301	18.8	1.8
Acetone	23.7	316	19.8	1.9
Hexane	18.4	246	15.4	1.5
Isopropanol	21.7	289	18.1	1.7

* Values are from Handbook of Physics and Chemistry, 66th Edition; ** Surface tension of water at *T* = 18 °C.

**Table 2:** Empirical correlations to predict diffusion coefficients for solutes in supercritical fluids at infinite dilution.

Reference	Equation
Funazukuri, Hachisu, and Wakao [62,67]	(7) $$ln\left(\frac{Sc-Sc^{o}}{Sc^{o}}\right)=-1.4ln\left(\frac{V_{2}-1.384{\left(V_{2}\right)}_{o}}{1.384{\left(V_{2}\right)}_{o}}\right)+1.48\;$$ $Sc=\frac{\eta_{2}}{\rho_{2}D_{12}}$ ${1.384(V_{2})}_{o}=4.452\times 10^{-5}-1.152\times 10^{-7}T+2.749\times 10^{-10}T^{2}-3.073\times 10^{-13}T^{3}+1.29\times 10^{-16}T^{4}$
	*V*_2_ is molar volume of solvent 2 (m^3^ mol^−1^), ${\left(V_{2}\right)}_{o}$ is solvent molar volume at which viscous flow stops, (m^3^ mol^−1^), *η* is visocity of solvent 2 (kg·m^−1^·s^−1^), *T* is absolute temperature (K), *Sc* is Schmidt number, $\rho_{2}$ is density of solvent 2 (kg·m^−3^). The superscript o indicates that the parameter is evaluated at atmospheric pressure.
He-Yu [68]	For $0.4\leq \rho_{r2}\leq 2.5,$ $0.66\leq T_{r2}\leq 1.78$ and $0.0581\leq M_{2}\leq 0.8854$ (8) $$D_{12}=10^{-10}\alpha \sqrt{\frac{T}{10^{3}M_{1}}\exp\left(\frac{0.3887V_{c2}}{V_{2}-0.23V_{c2}}\right)}$$ $\alpha =14.82+5.9081\left(\frac{T_{c2}V_{c2}}{M_{2}}\right)+2.0821{\left(\frac{T_{c2}V_{c2}}{M_{2}}\right)}^{2}$
	$\rho_{r2}$ is reduced density of solvent 2 (kg m^−3^), $M_{1\;}$ is molar mass of solute 1 (kg mol^−1^), $\;V_{c2}$ is critical molar volume of solvent 2 (m^3^ mol^−1^), $T_{c2}$ is critical temperature (K).
Funazukuri-Kong-Kagei [69,70]	(9) $$ln\left(\frac{Sc-Sc^{o}}{Sc^{o}}\right)=-4.92519817+54.5529385\left(\frac{{\left(V_{2}\right)}_{o}}{V_{2}}\right)-245.231443{\left(\frac{{\left(V_{2}\right)}_{o}}{V_{2}}\right)}^{2}+607.893924{\left(\frac{{\left(V_{2}\right)}_{o}}{V_{2}}\right)}^{3}-708.884016{\left(\frac{{\left(V_{2}\right)}_{o}}{V_{2}}\right)}^{4}+329.611433{\left(\frac{{\left(V_{2}\right)}_{o}}{V_{2}}\right)}^{5}$$ $Sc^{o}$ is calculated as: $Sc^{o}=\frac{5}{6}{\left(\frac{1}{2}+\frac{\sigma_{1}{}^{vdW}}{2\sigma_{2}{}^{vdW}}\right)}^{2}{\left(\frac{2M_{1}}{M_{1}+M_{2}}\right)}^{1/2}$ $\sigma_{1}{}^{vdW}={\left(\frac{6V_{i}{}^{vdW}}{\pi N_{av}}\right)}^{1/3}$
	${\left(V_{2}\right)}_{o}$ is molar volume of solvent 2 (m^3^ mol^−1^), T is absolute temperature (K), Sc is Schmidt number, $\sigma^{vdW}\;is\;Van\;der\;Waals\;diameter\;\left(m\right),$ N_av_ is avogadro number (6.022 $\times \;10^{23})$. The superscript *o* indicates that the parameter is evaluated at atmospheric pressure.
Eaton and Akgerman [66]	It is valid in the range 0.35 $\leq \rho_{r2}\leq$ 3.10 and 0.8 $\leq T_{r2}\leq 1.1$ $\rho_{r2}\;$ is reduced density of 2 (kg m^−3^) and $T_{r2}$ is reduced temperature of 2 (K).
	(10) $$D_{12}=1.42\times 10^{-21}\sqrt{T}\;{\left(\frac{\sigma_{1}^{eff}}{\sigma_{2}^{eff}}\right)}^{1.7538}{\left[\frac{M_{1}+M_{2}}{M_{1}M_{2}}\right]}^{1/2}\left(\frac{{\left(V_{2}\right)}_{o}}{{\left(\sigma_{12}^{eff}\right)}^{2}}\right)x\left[{\left(\frac{V_{2}}{{\left(V_{2}\right)}_{o}}\right)}^{e}-\frac{b_{12}}{{\left(V_{2}\right)}_{o}}\right]$$ $\frac{b_{12}}{{\left(V_{2}\right)}_{o}}=\left[-0.2440{\left(\frac{\sigma_{2}^{eff}}{\sigma_{1}^{eff}}\right)}^{2}+0.8491\left(\frac{\sigma_{2}^{eff}}{\sigma_{1}^{eff}}\right)+0.6001\right]\times {\left(\frac{M_{1}}{M_{2}}\right)}^{-0.03587}$ $e=\frac{\sigma_{2}^{eff}}{\sigma_{1}^{eff}}-\frac{1}{3}$ $\sigma_{i}^{eff}={\left(\frac{6V_{ci}}{\pi N_{av}}\right)}^{1/3}\;\left[0.552803-0.0026776T_{ri}\right]$ $\sigma_{12}^{eff}=\frac{\sigma_{1}^{eff}+\sigma_{2}^{eff}}{2}\;{\left(V_{2}\right)}_{o}=\frac{N_{av}\left(\sigma_{2}^{eff}\right)}{2^{1/2}}$ $\sigma^{eff}\;is\;molecular\;effective\;diamater\;\left(m\right).$

**Table 3:** Literature data on binary diffusion coefficient of various systems at infinite dilution.

System	*D*_21_ (10^−9^ m^2^ s^−1^)	Method
CO_2_ + ethanol at 25 °C	4.04	Diaphragm cell [72]
CO_2_ + ethanol at 25 °C	4.5	Wetted wall column [71]
CO_2_ + ethanol at 25 °C	4.11	Taylor dispersion [71]
CO_2_ + acetone at 30 °C	6.08	Diaphragm cell [73]
CO_2_ + methanol at 30 °C	4.95	Diaphragm cell [64]
CO_2_ + methanol at 25 °C	5.55 ± 0.09	Taylor dispersion [73]

**Table 4:** Literature data on effective diffusion coefficients in aerogels.

Sample	*D_e_* (10^−9^ m^2^ s^−1^)
Silica aerogel	5.75
IPA-CO_2_ system at 37.5 °C and 80 bar [31]	
Silica gel	3.05–5.52
Ethanol-CO_2_ system in the range from 20 °C to 42 °C at 90 bar [27]	
Silica aerogel	4.7–5.1
Ethanol-CO_2_ system at 40 °C 100 bar [5]	
Silica aerogel	5.5
Ethanol-CO_2_ system at 42 °C and 90 bar [46]	

**Table 5:** Mass transfer coefficient correlations.

Sherwood Number Correlations	Ref.
$Sh=2+1.1Re^{0.6}Sc^{1/3}$	[77]
$Sh=0.206Re^{0.8}Sc^{1/3}$	[75]
$Sh=0.38Re^{0.83}Sc^{1/3}$	[78]
$Sh=0.269Re^{0.83}Sc^{1/3}$	[74]
